# Mining of Potential Gene Resources for Breeding Nutritionally Improved Maize

**DOI:** 10.3390/plants11050627

**Published:** 2022-02-25

**Authors:** Quancan Hou, Tianye Zhang, Kangtai Sun, Tingwei Yan, Linlin Wang, Lu Lu, Wei Zhao, Yuchen Qi, Yan Long, Xun Wei, Xiangyuan Wan

**Affiliations:** 1 Research Center of Biology and Agriculture, Zhongzhi International Institute of Agricultural Biosciences, Shunde Graduate School, University of Science and Technology Beijing (USTB), Beijing 100024, China; houquancan@ustb.edu.cn (Q.H.); s20200906@xs.ustb.edu.cn (T.Z.); b20190395@xs.ustb.edu.cn (T.Y.); s20200896@xs.ustb.edu.cn (L.W.); g20198927@xs.ustb.edu.cn (L.L.); b20190396@xs.ustb.edu.cn (W.Z.); m202110892@xs.ustb.edu.cn (Y.Q.); 2 Beijing Engineering Laboratory of Main Crop Bio-Tech Breeding, Beijing Solidwill Sci-Tech Co., Ltd., Beijing International Science and Technology Cooperation Base of Bio-Tech Breeding, Beijing 100192, China; 3 Agricultural High-Tech Department, China Rural Technology Development Center, Ministry of Science and Technology, Beijing 100045, China; sunkt@pku.edu.cn

**Keywords:** maize, nutrient improvement, homologous gene, biological engineering

## Abstract

Maize is one of the leading food crops and its kernel is rich in starch, lipids, protein and other energy substances. In addition, maize kernels also contain many trace elements that are potentially beneficial to human health, such as vitamins, minerals and other secondary metabolites. However, gene resources that could be applied for nutrient improvement are limited in maize. In this review, we summarized 107 genes that are associated with nutrient content from different plant species and identified 246 orthologs from the maize genome. In addition, we constructed physical maps and performed a detailed expression pattern analysis for the 246 maize potential gene resources. Combining expression profiles and their potential roles in maize nutrient improvement, genetic engineering by editing or ectopic expression of these genes in maize are expected to improve resistant starch, oil, essential amino acids, vitamins, iron, zinc and anthocyanin levels of maize grains. Thus, this review provides valuable gene resources for maize nutrient improvement.

## 1. Introduction

**Figure 1:**
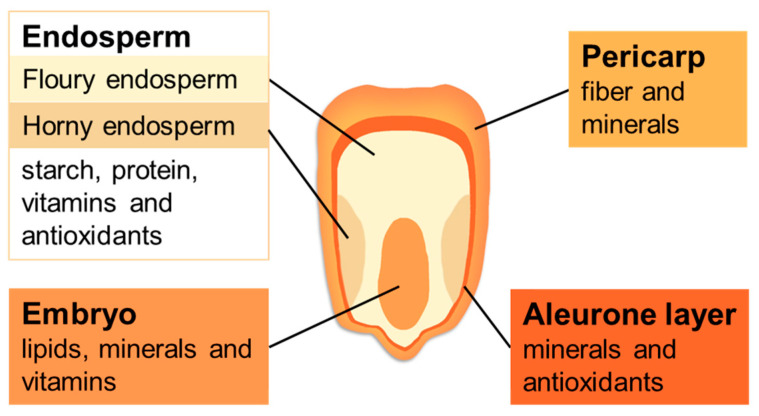
Structure and nutrient distribution of maize kernels.

Maize (*Zea mays* L.) is one of the main food crops in the world, which stands first among the grain crops in terms of yield production. In addition to being used as food for humans, maize can also be used as animal feed or as raw material for industrial manufacturing. Maize kernels provide many nutrients, including starch, oil and protein, and are rich in microelements, such as vitamins and minerals. The pericarp has fiber and minerals; the aleurone layer contains high levels of minerals and antioxidants; the endosperm contains starch, protein, vitamins and antioxidants; and the embryo is rich in lipids, minerals and some vitamins [1]. Understanding the distribution of various nutrients facilitates the application of appropriate methods to obtain desired components (Figure 1).

In face of the ever-changing demand for maize in the new era, traditional breeding strategies have the challenge of meeting human beings’ demands, from yield improvement to nutritional quality improvement. Compared with traditional breeding methods, molecular breeding significantly shortens the breeding process and has attracted more and more attention. Genome engineering technologies, including the CRISPR-Cas9 based genome editing and ectopic expression of functional genes driven by strong or tissue-specific promoters, have paved the way for molecular breeding [2,3]. So far, maize has the largest number of transgenic events that have been commercialized [4], which reflects the fact that maize improvement has attracted considerable attention and that genome engineering is profoundly changing the past and future of maize. Homologous genes among different plant species are highly likely to have similar functions. Many genes in model plant species such as rice (*Oryza sativa*) and *Arabidopsis thaliana* have been known to control specific traits. However, their homologs have not been identified or studied in maize. Therefore, delivering knowledge from model species to maize would be a rapid way to deliver maize nutrient improvement.

In this review, we focus on increasing the content of resistant starch, maize oil, essential amino acids, vitamins, minerals of iron and zinc, which are essential nutrients for human health. We summarize 107 genes that have been reported to be related to the above nutrient contents from different plant species, including *A. thaliana*, rice, soybean and potato, tomato, etc.; the protein sequences of these were used as queries to blast a maize genome with blastP on the Gramene website (http://ensembl.gramene.org/Zea_mays/Info/Index, accessed on 20 February 2022). All obtained sequences a with low *E*-value (<10^−12^) were selected for manual inspection. The Pfam domain searches (http://pfam.xfam.org/, accessed on 20 February 2022) were performed to confirm the candidate sequences as maize homologs. In addition, chromosomal mapping of these genes was carried out according to their positions on the chromosomes (Figure S1). Using available RNA_seq data, we analyzed the expression patterns of the 246 maize potential gene resources in maize early seeds, kernels and non-seed tissues. In addition, we also discuss the strategies of using these genes to obtain desired traits, providing a valuable candidate gene pool for nutrient improvement in maize.

## 2. Identification of Maize Potential Gene Resources for Starch Content Improvement

Starch accounts for most of the dry weight of corn kernels and provides calories for humans and animals. Starch comprises two types of polysaccharide molecules: amylose (Am) and amylopectin (Ap). Am is a polysaccharide made of D-glucose units, almost all of which are linked by α-1,4-glycosidic bonds, while Ap molecules are linked by α-1,4-glycosidic bonds and α-1,6-glycosidic bonds [5]. Four major enzymes are involved in starch synthesis. ADP-glucose, a glucosyl donor for starch synthesis, is synthesized by the catalyzation of adenosine diphosphate glucose pyrophosphorylase (AGPase), using glucose-1-phosphate (G-1-P) and ATP as substrates. Starch synthases (SSs) and starch branching enzymes (SBEs) are responsible for elongating the glucose polymer and branching, respectively. Debranching enzymes (DBEs) catalyze the hydrolysis of α-1,6-branch linkages of starch and other branched polyglucans, and an isoamylase-type (ISA) debranching enzyme facilitates the crystallization of amylopectin by hydrolyzing some of the branches and thus is involved in amylopectin synthesis [6].

A type of starch, known as resistant starch, cannot be digested by the stomach and small intestine where it can be fermented by certain specialized microorganisms [7]. Resistant starch plays an essential role in human health, including lowering blood glucose and cholesterol levels [8]. The proportion of Am in starch was found to positively correlate with resistant starch content in sorghum [9]. Thus, improvement of Am content also indirectly increases the content of resistant starch.

Am content in maize kernels could be adjusted by altering the direction of starch synthesis. According to the starch synthesis process described above, SBE is the most critical factor in converting Am and Ap. Studies on rice [10,11], wheat [12], barley [13], potatoes [14,15] and cassava [16] showed the content of Am was increased when the activity of SBEs was suppressed, supporting the notion that manipulation of SBE is an effective way to increase the Am content. The effect of SSs on starch synthesis has been investigated and confirmed in rice [17,18] and sweet potatoes [19]. Granule bound starch synthase (GBSS) binds specially to starch and maintains the unbranching state of Am while the Protein Targeting to Starch 1 (PTST1) participates in the localization of GBSS in Am. Studies showed that boosting the expression of *GBSS* and *PTST1* resulted in the enhancement of Am production [20,21,22]. Thus, we assume that harnessing these key enzymes involved in starch synthesis could also effectively improve the starch content in maize kernels. Sixteen homologous genes encoding these key enzymes were identified from the maize genome (Table 1).

## 3. Identification of Maize Potential Gene Resources for Oil Content Improvement

Corn oil is a byproduct of corn wet-milling industries, and is a significant part of the human diet, useful in industrial applications and an alternative to fossil fuels. Corn oil is mainly composed of 59% polyunsaturated (PUFA), 24% monounsaturated (MUFA) and 13% saturated fatty acid (SFA) [23]. To enhance the economic value of corn, genome engineering is efficient and effective in improving the oil content of corn kernels [24]. Many enzymes, carrier proteins and transcription factors (TF) associated with the regulation of oil yield have been identified in other species. Genes encoding these proteins are potential resources for generating high oil-yielding maize by genome engineering.

**Figure 2:**
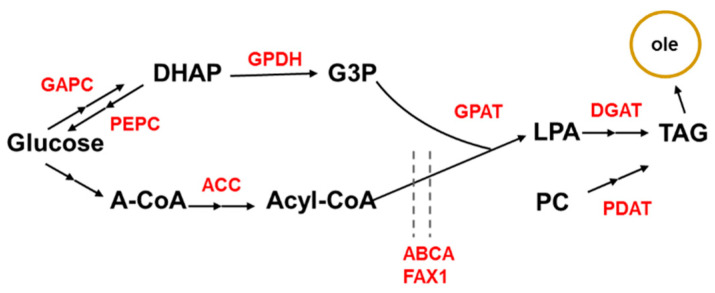
Schematic representation of the TAG biosynthetic pathway in plants. (GAPC, glyceraldehyde-3-phosphate dehydrogenase; PEPC, phosphoenolpyruvate carboxylase; DHAP, dihydroxyacetone phosphate; GPDH, Glycerol-3-phosphate dehydrogenase; G3P, glycerol-3-phosphate; A-CoA, acetyl-CoA; ACC, acetyl-CoA carboxylase; GPAT, glycerol-3-phosphate acyltransferase; LPA, lysophosphatidic acid; DGAT, diacylglycerol acyltransferase; PC, phatidylcholine; PDAT, phospholipid diacylglycerol acyltransferase; TAG, triacylglycerol). Dash lines represent transmembrane transport.

The chemical composition of oil is triacylglycerol (TAG) formed from the sequential acylation of three fatty acids (FAs), with glycerol-3-phosphate (G3P) as the backbone. TAG de novo synthesis is catalyzed in the Kennedy pathway and is affected by the glycolysis and tricarboxylic acid cycle (TCA) processes. Glyceraldehyde-3-phosphate dehydrogenase (GAPC) catalyzes the reaction of glyceraldehyde-3-phosphate to 1,3-bisphosphoglycerate, and phosphoenolpyruvate carboxylase (PEPC) catalyzes the reaction of oxaloacetic acid to phosphoenolpyruvate. Overexpression of *GAPC* or silencing of *PEPC* promotes glycolysis and indirectly increases the content of dihydroxyacetone phosphate (DHAP) [25,26]. Glycerol-3-phosphate dehydrogenase (GPDH) converts DHAP into glycerol-3-phosphate (G3P). Acetyl-CoA, a product of TCA, is promoted to malonyl-CoA by acetyl-CoA carboxylase (ACC), which enters the Kennedy pathway together with G3P. Glycerol-3-phosphate acyltransferase (GPAT) catalyzes G3P into lysophosphatidic acid (LPA), which is the first step in glycerolipid biosynthesis [27]. LPA or phatidylcholine (PC) are further catalyzed by a series of enzymes, including diacylglycerol acyltransferase (DGAT) and phospholipid diacylglycerol acyltransferase (PDAT), and finally form TAG (Figure 2). Studies have shown that GPAT [28], DGAT [29,30,31] and PDAT [30] directly affect the TAG synthesis. Transcription factors of AtMYB89 [32], AtMYB96 [33], LEC [34,35,36,37],GL2 [38,39], FUS3 [40] and HB2 [41] are involved in TAG biosynthesis regulation.

Fatty acids are initially generated in the plastid and are transported to the endoplasmic reticulum for TAG synthesis. FAX1 and ABCA9 are identified as the carrier proteins for fatty acid transport from the plastid to the endoplasmic reticulum. In addition, *Oleosin* (*OLE*) encodes the most abundant seed oil droplet-specific protein, overexpression of which increases oil levels in rice and soybean [30,42]. On the other hand, silencing of *sugar dependent 1* (*SDP1*), which inhibits the degradation of TAG, could also lead to increases in TAG content [31,43,44]. The TAG synthesis pathway and key enzymes are shown in Figure 2. Sixty-one homologous genes involved in TAG synthesis were identified from the maize genome (Table 2).

## 4. Identification of Maize Potential Gene Resources for Essential Amino Acid Content Improvement

**Figure 3:**
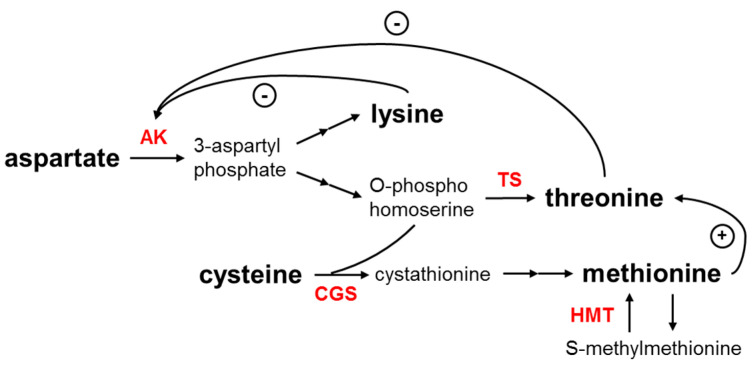
Schematic representation of amino acid biosynthetic pathways in plants. Curved arrows with a (−) sign represent major feedback inhibition loops by the end product amino acids and arrows with a (+) sign represent activation. (AK, aspartate kinase; TS, threonine synthase; CGS, cystathionine γ-synthase; HMT, homocysteine S-methyltransferase).

Essential amino acids are vital for protein synthesis, tissue repair and nutrient absorption. For instance, both lysine and tryptophan are important components of neurotransmitters. However, humans and animals cannot synthesize essential amino acids and can only get them from diets rich in proteins. Grains are low in lysine, while beans are poor in methionine. In maize kernels, protein content ranges from 7% to 14%, depending on genotype and environmental effects [1]. Here, we focus on improving the content of three important essential amino acids: methionine, lysine and tryptophan, as well as total protein (Figure 3).

Both lysine and methionine are synthesized from aspartate via different pathways. Aspartate kinase (AK) catalyzes the first step, which is a rate-limiting step that requires ATP, and this step is also regulated by subsequent steps in a feedback manner. Single amino acid substitution mutants of *ak* are insensitive to the feedback inhibition of lysine synthesis and elevate lysine content [52]. Asparaginyl-tRNA synthetase (SYNC) mediates the process of linking amino acids to tRNAs, and over-accumulation of the SYNC has been shown to increase lysine levels [53]. Increasing the abundance of proteins rich in lysine, such as VSP and BiP, elevates lysine content [54,55,56]. Cystathionine γ-synthase (CGS), the first specific enzyme for methionine synthesis using cysteine as a precursor, is critical for the control of methionine content [57,58]. The backflow from S-methylmethionine to methionine is mainly catalyzed by homocysteine S-methyltransferase (HMT). Threonine synthase (TS) is a key enzyme for threonine synthesis but affects methionine formation as it competes with TS for their common substrate O-phosphohomoserine. More TS makes the resource flow to threonine, which is not conducive to the formation of methionine [59]. Studies have shown that mutations in *ts* boost the methionine level [60,61]. In the pathway of tryptophan synthesis, anthranilate synthase (ASA) is a key enzyme of the process and affects the pathway [62]. Aspartate aminotransferase (AAT) is also one of the important targets for improving protein content as it participates in the regulation of carbon and nitrogen metabolism through the transfer of the amino group [63]. Asparagine synthetase (ASN) has a similar function to AAT. Carrier protein SUT1 [64], AAP6 [65] and TF TaNAC100 [66] were also found to affect the protein content of grains. Twenty-eight homologous genes involved in essential amino acid biosynthesis were identified from the maize genome (Table 3).

## 5. Identification of Maize Potential Gene Resources for Vitamin Content Improvement

After decades of relentless efforts by plant breeders, the yield of staple crops has increased dozens of times. However, hidden hunger, which refers to eating food that is insufficient in vitamins and micronutrients, becomes a new problem that afflicts more than 2 billion people globally. Crops such as corn are unable to provide sufficient micronutrients and need to undergo biofortification, which uses agricultural methodologies to augment the nutritional quality of food and counter micronutrient malnutrition [71]. Vitamins are essential micronutrients for growth, metabolism, reproduction and other processes related to human health. Vitamins can be classified into two groups: fat-soluble (A, D, E and K) and water-soluble (B and C).

β-carotene is a kind of red-orange pigment, which imparts color and antioxidant properties to plants and fruits. When it enters the body, it turns into vitamin A. Deficiency in vitamin A can cause night blindness. Isoprenoids produced by the 2-C-methyl-D-erythritol 4-phosphate (MEP) pathway are carotenoid precursors [72]. 1-deoxy-D-xylulose 5-phosphate synthase (DXS) and 1-Deoxy-D-xylulose 5-phosphate reductoisomerase (DXR) are important catalytic enzymes for β-carotene synthesis. Phytoene desaturase (PDS) also promotes the formation of carotene. In addition to improving the synthesis efficiency through overexpressing key enzymes, some other factors that are not directly involved in the biosynthetic pathway have been shown to affect carotenoid accumulation in several plant species. Ectopic expression of ORANGE (OR), a plastidial DnaJ cysteine-rich domain-containing protein governing chromoplast biogenesis and carotenoid accumulation, promotes carotenoid accumulation and fruit development in tomatoes [73,74]. Suppression of de-etiolated 1 (det1) that affects plant light absorption via RNAi alters the carotenoid content in tomatoes and *brassica napus* [75,76]. Ectopic expression of a brassinazole-resistant 1 (bzr1–1d) transcription factor in brassinosteroid signaling enhances carotenoid accumulation in tomatoes [77]. Transcription factors cytosine-mismatch-binding protein 1 (CMB1) and stay-green protein (SGR1) were found to regulate carotenoid accumulation during fruit ripening in tomatoes [78,79]. As an antioxidant, β-carotene is easily degraded by light, heat and oxygen. The inhibition of carotenoid cleavage dioxygenase (CCD) and lipoxygenase (LOX) can delay the degradation of β-carotene [80,81,82].

For mammals, deficiency in vitamin E is associated with some cancers, as well as neurodegenerative and cardiovascular diseases. Vitamin E is made up of four tocopherols and four tocotrienols, of which α-tocopherol is the most active form. The phytol moiety of tocopherols could be derived from chlorophyll. Chlorophyll dephytylase (CLD) and chlorophyll synthase (CHLG) are involved in the synthesis and decomposition of the process, respectively. ρ-hydroxyphenylpyruvate dioxygenase (HPPD), homogentisate phytyltransferase (HPT), homogentisic acid geranylgeranyl transferase (HGGT), 2-methyl-6-phytylbenzoquinol methyltransferase (MPBQMT) and tocopherol cyclase (TC) participate in the next catalytic steps for tocopherol formation. Overexpression of these enzyme encoding genes is conducive to the synthesis of tocopherols. Studies have found that tocopherol-binding protein (TBP) is a transporter of tocopherols and that silencing of TBP reduces the content of tocopherols [83].

Vitamin C, also known as L-ascorbic acid, is a water-soluble vitamin and plays important roles in supporting cardiovascular function, immune cell development, iron utilization and other functions. Although ascorbic acid is an important antioxidant, it cannot be synthesized by humans and must be obtained from food. Many methods have been developed to increase the amount of ascorbic acid, and some of them have already been applied to maize. The synthetic reaction of ascorbic acid originates from gluctose-6-phosphate (G6P); G6P is transformed into GDP-mannose through a series of enzymatic reactions, and then by the catalyzation of GDP-mannose 3,5-epimerase (GME), GDP-galactose phosphorylase (GGP), galactose-1-phosphate phosphatase (GPP), galactose dehydrogenase (GDH) and galactono-1,4-lactone dehydrogenase (GalLDH), finally forming ascorbic acid. All of these enzymes have been shown to contribute to synthetic reactions. Animals and plants synthesize ascorbic acid through completely different pathways but both use L-gulono-1,4-lactone oxidase (GulLO). Therefore, GulLO is a common enzyme that can boost the ascorbic acid content both in animals and plants [84]. Besides, dehydroascorbate reductase (DHAR) can facilitate ascorbic acid regeneration [85,86].

**Figure 4:**
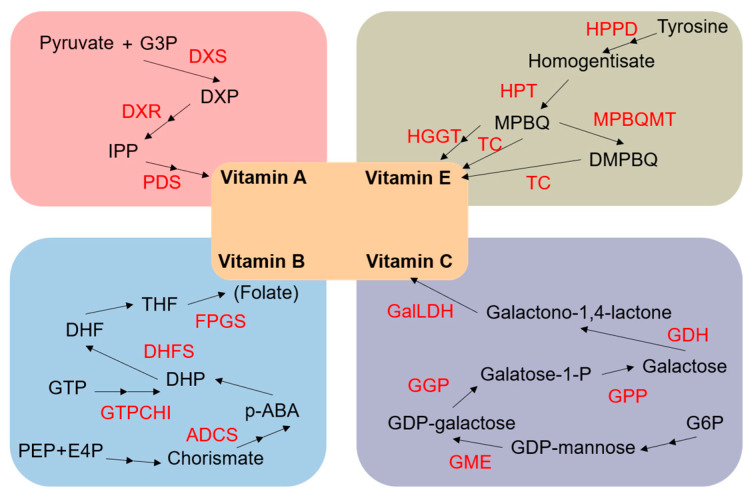
Schematic representation of vitamin biosynthetic pathways in plants. (G3P, glycerol-3-phosphate; DXS, 1-deoxy-D-xylulose 5-phosphate synthase; DXP, 1-deoxy-D-xylulose-5-phospate; DXR, 1-Deoxy-D-xylulose 5-phosphate reductoisomerase; IPP, isopentenyl diphosphate isomerase; PDS, phytoene desaturase; HPPD, ρ-hydroxyphenylpyruvate dioxygenase; HPT, homogentisate phytyltransferase; MPBQ, methylphytylbenzoquinol; HGGT, homogentisic acid geranylgeranyl transferase; TC, tocopherol cyclase; MPBQMT, 2-methyl-6-phytylbenzoquinol methyltransferase; DMPBQ, dimethylphytylbenzoquinone; PEP, phosphoenolpyruvate; E4P, erythrose 4-phosphate; ADCS, aminodeoxychorismate synthase; p-ABA, para-aminobenzoate; DHP, dihydropteroate; GTPCHI, GTP cyclohydrolase I; DHFS, dihydrofolate synthetase; DHF, dihydrofolate; THF, tetrahydrofolate; FPGS, folylpolyglutamate synthase; G6P, glucose-6-phosphate; GME, GDP-mannose 3,5-epimerase; GGP, GDP-L-galactose phosphorylase; GPP, L-galactose-1-phosphate; GDH, L-galactose dehydrogenase; GalLDH, L-galactono-1,4-lactone dehydrogenase).

Group B vitamins are a set of enzyme cofactors, and their derivatives include thiamin, riboflavin, niacin, pantothenate, pyridoxine, biotin, folate, cobalamin and so on. Group B vitamins also play a critical role in human health, coordinating the metabolism of the body, but their mechanisms are not well understood. In the case of folate, GTP is its synthetic substrate, which is first catalyzed by GTP cyclohydrolase I (GTPCHI). After entering the mitochondrion, it merges with para-aminobenzoate (p-ABA) from plastid and is catalyzed by aminodeoxychorismate synthase (ADCS) and other enzymes. Overexpression of two vital enzymes, dihydrofolate synthetase (DHFS) or folylpolyglutamate synthase (FPGS), improves the efficiency of folate synthesis [87]. These enzymes add glutamate to folate and increase its stability, while γ-glutamyl hydrolase (GGH) hydrolyzes it. On the contrary, overexpression of GGH decreases the level of folate [88]. The key enzymes involved in vitamin A, B, C and E synthesis are shown in Figure 4. Fifty-six homologous genes involved in vitamin synthesis were identified from the maize genome (Table 4).

## 6. Identification of Maize Potential Gene Resources for Mineral Content Improvement

Minerals can be used as the components of some special substances in the human body and also as a co-enzyme to participate in metabolism as well as maintain cell membrane permeability and other various functions. It is important to understand mineral transport processes, since the minerals within food need to be taken up by plants from the soil. Plants have evolved two strategies for iron absorption. In dicots, Fe^3+^ is reduced to Fe^2+^ and then transported into cells. Unlike this, grass plants, such as maize, could directly chelate Fe^3+^ by mugineic acid (MA) for transport. However, rice uses both strategies for iron uptake [97]. Overexpression of nicotianamine synthase (NAS) and nicotianamine aminotransferase (NAAT), both of which participate in MA biosynthesis, facilitates the transport of iron [98,99]. NRAMP1 and NRAMP5 are two important transporters responsible for transporting Fe from roots to above-ground tissues where Fe could be stored in seeds. Fe chelates with citrate as it flows through the vascular, and FRD3 and FRDL1 are involved in the citrate transport. Fe is stored in vacuoles in plants, and VIT1 and NRAMP4 are responsible for the positive and negative regulation of vacuolar Fe content, respectively. Vacuolar Fe stores can be used to increase endosperm Fe content by inhibiting or promoting the expression of VIT1 and NRAMP4, respectively [100,101]. Overexpression of endosperm Fe storage protein FER significantly increases Fe content in the endosperm [102]. Many TFs have been found to regulate Fe uptake and transport from different plant species, including IRO2 [103], OsbHLH58 [104], OsbHLH59 [104], AtbHLH29 [105], GmbHLH300 [106], IDEF1 [107] and CSN6 complex [108]. In addition, Rab6a, as a subunit of small GTPase, is involved in adaption to CO_2_ enrichment, thereby regulating photosynthesis and Fe content [109]. Fe-binding ubiquitin ligase (HRZ) is associated with the negative regulation of the Fe transport pathway [110]. Zn transport is similar to Fe, and overexpression of NAS and NAAT also increases Zn content. However, many transporters are unique for Zn transport; these include MTP1 [111], ZIF1 [112], ZIF2 [113], HMA2 [114], HMA4 [115], HMA7 [116], ZIP1 [117], ZIP8 [118] and so on.

Despite the fact that improving the transport efficiency of microelements could increase their contents in plants, plants contain a special anti-nutrient myo-inositol 1,2,3,4,5,6-hexakisphosphate (InsP6), commonly known as phytic acid (PA), which seriously affects human absorption of minerals. PA strongly chelates cations to form phytate, an insoluble salt that blocks the absorption of Fe and Zn from the human gut. The first step in the PA synthesis pathway is the conversion of glucose-6-phosphate to myo-inositol-3-phosphate by myo-inositol-1-phosphate synthase (MIPS), following which the myo-inositol-3-phosphate is further phosphorylated by 2-phosphoglycerate kinase (PGK), inositol 1,3,4-trisphosphate 5/6-kinase (ITPK) and inositol 1,3,4,5,6-pentakisphosphate 2-kinase (IPK), to finally form PA [119]. PA could be downregulated by either inhibiting the production of these enzymes or promoting the synthesis of phytases such as HAD and PAP. Seventy-six homologous genes involved in mineral absorption, transport and regulation were identified from the maize genome (Table 5).

## 7. Identification of Maize Potential Gene Resources for Other Secondary Metabolites Content Improvement

In addition to vitamins, there are many secondary metabolites in plants, mainly phenolic compounds. The majority of the phenolic compounds in maize are phenolic acids, such as ferulic, vanillic, caffeic, syringic, synaptic and ρ-coumaric acids, and polyphenols, including lignins and lignans [131]. Phenolic compounds are essential for plant growth and development and are considered as defensive barriers of plants. However, the detailed mechanism is still unknown, and it is speculated that it plays an antioxidant role [132].

Anthocyanins are flavonoids that confer plant seeds and fruits various colors, from red to purple. They are not just protective agents for plants. Anthocyanins are also used as supplements in health care products to control obesity and diabetes and improve vision and brain function [1]. Many genes related to anthocyanin synthesis have been identified and applied in genetic engineering to improve anthocyanin content in maize. These include many transcription factors that regulate anthocyanin synthesis. For example, GLK1 [133], AN1 [134], AN3 [135] and ANT1 [136] are positive regulators, while GmMYBR [137] is a negative regulator for anthocyanin synthesis. A double-stranded RNA binding protein, DRB3 has also been shown to inhibit anthocyanins biosynthesis [138]. Nine homologous genes involved in anthocyanin synthesis were identified from the maize genome (Table 6).

## 8. Expression Patterns of the Putative Nutritional Improvement-Related Maize Genes

**Figure 5:**
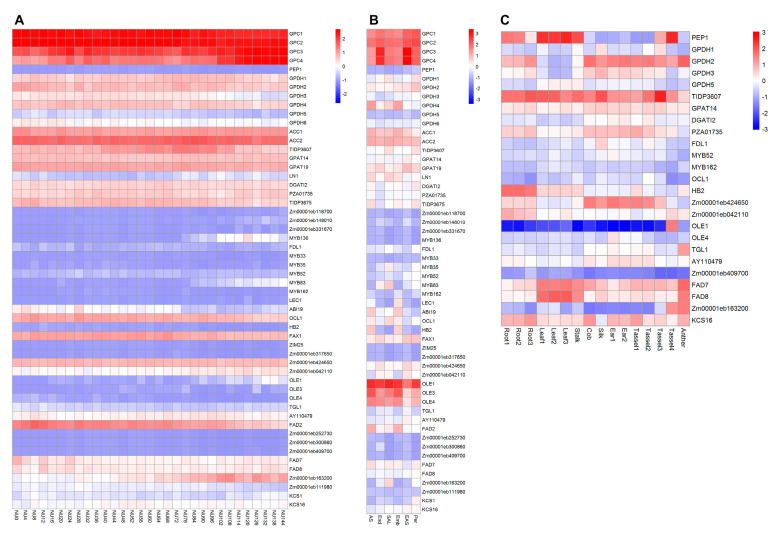
Expression profiles of potential gene resources for maize lipid content improvement. (**A**) Expression heatmap of potential gene resources for maize lipid content improvement from maize nucellus at different time points after pollination. NU0-144 represents the nucellus (embryo sac included) after 0–144 h of self-pollination. (**B**) Expression heatmap of potential gene resources for maize lipid content improvement from maize kernels. AS, Apical scutellum; End, Endosperm; SAL, Scutellar Alleurone Layer; Emb, Embryo; EAS, Endosperm Adjacent to Scutellum; Per, Pericarp. (**C**) Expression heatmap of potential gene resources for maize lipid content improvement from 16 maize tissues. Root1,2,3 represent V2, V5 seminal root and adult nodal root, respectively; Leaf1,2,3 represent the 2nd, 4th and 8th leaf, respectively; Ear1,2 represent V8 and V15 ear, respectively; Tassel 1,2,3,4 represent 1 mm, 2 cm, 12 cm and 22 cm tassel, respectively. The color scale bars represent the relative expression level.

For expression analysis of identified potential gene resources, we used 31 different time points seed samples [139] and 6 kernel compartment samples [140] of the B73 inbred line of the RNA-seq data and 16 non-seed tissues of the inbred line SRG200 (Syngenta) of the microarray data [141] downloaded from the Maize eFP database (https://bar.utoronto.ca/efp_maize/cgi-bin/efpWeb.cgi?, accessed on 20 February 2022). The expression levels of each gene in different tissue are listed in Table S1, and gene expression heatmaps were generated using the pheatmap package of R software (Figure 5 and Figure S2). Most identified genes are highly expressed in either early seeds or kernels. However, some genes are weakly expressed in both tissues. For instance, *GPC1*, *GPC2*, *GPC3* and *GPC4* that encode glyceraldehyde-3-phosphate dehydrogenases are highly expressed in nucellus at different time points after pollination and in different compartments of kernels. *OLE1*, *OLE3* and *OLE4* that encode delta-9 desaturases are weakly expressed in early seeds but highly expressed in different compartments of kernels. This may be because OLEs play a structural role in stabilizing the lipid body during desiccation of the seed by preventing coalescence of the oil (Figure 5). The expression profiles of the putative genes provide important information for the strategy applied to the molecular breeding of nutritionally enriched maize.

## 9. Discussion

In this review, we summarized genes associated with nutrient biosynthesis, uptake and transport from different plant species, and 246 homologous genes were identified from the maize genome. These genes are promising candidates for improving resistant starch, oil, essential amino acids, vitamins, iron, zinc and anthocyanin levels of maize grains through genome engineering. However, one should also notice that plant phylogeny is complex, and the function of a gene cannot be completely determined from homology alone. Therefore, information regarding maize kernel transcriptome and metabolome would be helpful for the validation of the candidate genes for breeding use. Metabolic profiling of mature maize kernels revealed significant variation among different maize lines. For example, glucose-1-phosphate (G1P) is an intermediate in starch metabolism and was identified as the highest variable metabolite between maize varieties Chang7-2 and Ye478 [142]. UDP-Glycosyltransferase super family proteins catalyze G1P into glucuronate as annotated in the KEGG database. *Zm00001eb214570* is an ortholog of *AT3G02100* in *A. thaliana* and encodes a UDP-Glycosyltransferase. The expression of *Zm00001eb214570* was undetectable in Ye478 [143] and the level of glucuronate was much lower in Ye478 compared to Chang7-2. Therefore, the accumulation of a high level of G1P in Ye478 probably results from the lack of the expression of *Zm00001eb214570*. This indicates that metabolomics is generally correlated with transcriptomics. However, one should note that even if the prediction of a gene’s functions is reliable and correct, changes in the metabolic rate of an intermediate process may not have a significant effect on the amount of material synthesized, as precursor materials limit the final content.

The key enzyme SSs for maize starch synthesis are encoded by many homologous genes, which probably have function redundancy. In rice, the repression of genes that encode isozymes SSI, SSIIa and SSIIIa via RNAi strongly influenced grain development, while repression of the other four SS encoding genes did not show any effect [17]. Another study on rice *SS* has also suggested that improved grain quality can only be achieved by coordinated downregulation of the expression of SSIIb and SSIIc, indicating a functional redundancy between SSIIb and SSIIc [18].

Previous studies mostly focused on breeding high-yield oil-corn varieties for industrial use. Recently, more and more attention has been paid to improving the nutritional properties of corn. Studies have shown that unsaturated fatty acids are better for human health than saturated fatty acids. In order to reduce the amount of PUFA and increase the amount of oleic acid, a type of MUFA, delta-12 fatty acid desaturase 2 (FAD2), delta-12 fatty acid desaturase 3 (FAD3) and fatty acid elongase 1 (FAE1) are good targets for genetic manipulation. Inhibition of FAD2 [50,51], FAD3 [50] and FAE1 [51] increases the content of MUFA.

Another issue that should be addressed is that many genes have multiple functions and are expressed in various plant tissues and organs. Therefore, applying these genes for nutrient quality improvement using a knockout strategy may also cause serious plant growth and development defects, and the knockdown strategy may be more suitable for such cases. Additionally, protein engineering to generate amino acid substitution mutants instead of knockout may provide another option to solve this problem. This requires knowledge of the working mechanism of the protein and the specific mutation technology.

The mechanisms of vitamin synthesis, mineral absorption and transport are still not fully clear. Many vitamins need to work together, so multivitamins are now advocated. Vitamin absorption also has a great relationship with the cooking method. Studies have shown that the cooking temperature was the decisive factor in the cooking loss of carotenoids in corn, and the boiling and steaming of corn caused it to retain the most nutrients [144]. Using exogenous fertilization seems more straightforward for mineral replenishment, but the cost and problems associated with soil and groundwater contamination make genetic manipulation a better choice. However, the effect of genetic manipulation for mineral content improvement is also related to the cultivar. Although phytic acid is harmful to the absorption of metal ions, it is also the storage form of phosphorus in plants. In order to avoid the effect on phosphorus uptake, the regulation of phytic acid content should be carefully considered.

In addition to modifying plant genes, genetic engineering allows the possibility of introducing genes with special effects from other species such as bacteria into maize. The use of zein promoters that specifically express bacterial *crtB* and *crtI* genes in maize endosperm resulted in a thirty-four times increase in total carotene [145]. The bacterial *lysC* gene encodes an AK, but unlike AK in plants, it is not inhibited by lysine feedback, so when *lysC* was ectopically expressed in tobacco seeds, lysine content was increasingly detected [146]. Similar strategies could also be applied to maize.

Many genes show synergistic effects on a specific biological process. Therefore, overexpression of a series of synthases along the same synthetic pathway may cause more substantial effects than overexpression of one gene alone. Some genes may regulate a synthetic pathway coordinately. For example, when *HGGT* is co-expressed with carotene synthesis genes in sorghum, increased vitamin E can reduce the oxidative degradation of carotene, increase the stability and half-life of carotene and thus increase the carotene content [89].

## 10. Conclusions

With the increasing population and human nutritional requirements for the daily diet, developing nutrient-rich high-yield crop varieties has become breeders’ primary objective. Biofortification is a good way to improve the nutrient content of plants, and there is much room for application in maize. The development of transcriptomics and metabolomics has provided valuable information for disclosing mechanisms of nutrient compound synthesis. In this review, we summarized the reported genes that are associated with nutrient content from different plant species. Based on the principle that plant homologous genes may have similar functions across species, we identified 246 genes related to nutrient quality from the maize genome and provided physical maps for their chromosome location and detailed expression profiles in early seeds, kernels and non-seed tissues. These genes are potential resources for improving the content of starch, oil, protein, vitamin, mineral and secondary metabolites in maize kernels. Combining the data from transcriptomic, proteomic and metabolomic analyses, constructing maize kernels’ transcriptional, proteomic and metabolic roadmaps will provide a comprehensive relationship between gene regulation and metabolic network, which facilitates gene function validation and future maize breeding with the aim to improve nutritious quality.

## Figures and Tables

**Table 1:** List of potential gene resources for improving resistant starch content in maize.

Genes	Protein Function	Maize Orthologs	Gene ID	Strategy	References
*SBE*	starch branching enzyme	*SBE1*	*Zm00001eb228530*	knockout	[10,11,12,13,14,15,16]
		*SBE3*	*Zm00001eb357830*		
		*SBE4*	*Zm00001eb084160*		
		*AE1*	*Zm00001eb242610*		
*SS*	starch synthase	*SS1*	*Zm00001eb376100*	overexpression	[17,18,19]
		*SS2*	*Zm00001eb070230*		
		*SS3*	*Zm00001eb431240*		
		*SS4*	*Zm00001eb353810*		
		*SS5*	*Zm00001eb191890*		
		*SS6*	*Zm00001eb222830*		
		*SS7*	*Zm00001eb194550*		
		*DU1*	*Zm00001eb413290*		
		*SU2*	*Zm00001eb279740*		
*GBSS*	granule bound starch synthase	*WX1* *GBSS1*	*Zm00001eb378140* *Zm00001eb305810*	overexpression	[20,21,22]
*PTST1*	protein targeting to starch	*GPM177*	*Zm00001eb231700*	overexpression	[20,21]

**Table 2:** List of potential gene resources for enhancing lipid yield in maize.

Genes	Protein Function	Maize Orthologs	Gene ID	Strategy	References
*GAPC*	glyceraldehyde-3-phosphate dehydrogenase	*GPC1*	*Zm00001eb173410*	overexpression	[25]
		*GPC2*	*Zm00001eb261430*		
		*GPC3*	*Zm00001eb184000*		
		*GPC4*	*Zm00001eb246370*		
*PEPC2*	phosphoenolpyruvate carboxylase	*PEP1*	*Zm00001eb383680*	knockout	[26]
*GPDH*	glycerol-3-phosphate dehydrogenase	*GPDH1*	*Zm00001eb141610*	overexpression	[45]
		*GPDH2*	*Zm00001eb369390*		
		*GPDH3*	*Zm00001eb352530*		
		*GPDH4*	*Zm00001eb139850*		
		*GPDH5*	*Zm00001eb303710*		
		*GPDH6*	*Zm00001eb419210*		
*ACC1*	acetyl-CoA carboxylase	*ACC1* *ACC2* *TIDP3607*	*Zm00001eb419400* *Zm00001eb086560* *Zm00001eb223980* *Zm00001eb028920* *Zm00001eb189990* *Zm00001eb367400*	overexpression	[46,47]
*GPAT9*	glycerol-3-phosphate acyltransferase	*GPAT14*	*Zm00001eb396350*	overexpression	[28]
		*GPAT19*	*Zm00001eb323170*		
*DGAT1*	diacylglycerol acyltransferase	*LN1*	*Zm00001eb277490*	overexpression	[29,30,31]
		*DGATI2*	*Zm00001eb284200*		
*PDAT*	phospholipid diacylglycerol acyltransferase	*PZA01735* *TIDP3675*	*Zm00001eb100310* *Zm00001eb314300* *Zm00001eb118700* *Zm00001eb148010* *Zm00001eb331670* *Zm00001eb342120*	overexpression	[30]
*MYB89*	transcription factor	*MYB136*	*Zm00001eb128770*	knockout	[32]
*MYB96*	transcription factor	*FDL1*	*Zm00001eb328280*	overexpression	[33]
		*MYB33*	*Zm00001eb041330*		
		*MYB35*	*Zm00001eb099570*		
		*MYB52*	*Zm00001eb392230*		
		*MYB70*	*Zm00001eb109860*		
		*MYB83*	*Zm00001eb041320*		
		*MYB162*	*Zm00001eb312600*		
*LEC1*	transcription factor	*LEC1*	*Zm00001eb253260*	overexpression	[34,35]
*LEC2*	transcription factor	*ABI19*	*Zm00001eb361390*	overexpression	[36,37]
*GL2*	transcription factor	*OCL1*	*Zm00001eb126140*	knockout	[38,39]
*FUS3*	transcription factor	*ABI19*	*Zm00001eb361390*	overexpression	[40]
*HB2*	transcription factor	*HB2*	*Zm00001eb293010*	overexpression	[41]
*FAX1*	carrier protein	*FAX1* *ZIM25*	*Zm00001eb301150* *Zm00001eb379540* *Zm00001eb317650* *Zm00001eb424650*	overexpression	[48]
*ABCA9*	carrier protein		*Zm00001eb042110*	overexpression	[49]
*OLE*	delta-9 desaturase	*OLE1*	*Zm00001eb074940*	overexpression	[30,42]
		*OLE3*	*Zm00001eb216880*		
		*OLE4*	*Zm00001eb053890*		
*SDP1*	sugar dependent	*TGL1*	*Zm00001eb370460*	knockout	[31,43,44]
		*AY110479*	*Zm00001eb062080*		
*FAD2*	delta-12 fatty acid desaturase	*FAD2*	*Zm00001eb188990* *Zm00001eb252730* *Zm00001eb300860* *Zm00001eb409700* *Zm00001eb442020*	knockout	[50,51]
*FAD3*	delta-12 fatty acid desaturase	*FAD7* *FAD8*	*Zm00001eb397050* *Zm00001eb013340* *Zm00001eb163200* *Zm00001eb111980*	knockout	[50]
*FAE1*	fatty acid elongase	*KCS1*	*Zm00001eb344070*	knockout	[51]
		*KCS16*	*Zm00001eb296230*		

**Table 3:** List of potential gene resources for elevating essential amino acid content in maize.

Genes	Protein Function	Maize Orthologs	Gene ID	Strategy	References
*AK*	aspartate kinase	*ASK1*	*Zm00001eb064530*	knockout	[52]
		*ASK2*	*Zm00001eb094670*		
*SYNC1*	asparaginyl-tRNA synthetase		*Zm00001eb341390*	overexpression	[53]
*VSP*	storage protein	*VSP1*	*Zm00001eb283460*	overexpression	[54,55]
		*VSP2*	*Zm00001eb283450*		
*BIP*	storage protein	*BIP1*	*Zm00001eb229930*	overexpression	[56]
		*BIP2*	*Zm00001eb209550*		
		*BIP3*	*Zm00001eb214940*		
*CGS*	cystathionine γ-synthase	*CGS1*	*Zm00001eb392050* *Zm00001eb018300*	overexpression	[57,58]
*TS1*	threonine synthase	*THR1* *THR2* *THR3*	*Zm00001eb156020* *Zm00001eb294790* *Zm00001eb284240* *Zm00001eb022690* *Zm00001eb088230*	knockout	[60,61]
*HMT*	homocysteine S-methyltransferase	*HMT1*	*Zm00001eb399940*	overexpression	[67]
*ASA*	anthranilate synthase		*Zm00001eb063220* *Zm00001eb211420*	overexpression	[68,69]
*AAT*	aspartate aminotransferase	*GOT1*	*Zm00001eb152450*	overexpression	[63]
		*GOT2*	*Zm00001eb257910*		
		*GOT3*	*Zm00001eb238900*		
		*GOT4*	*Zm00001eb146400*		
*ASN1*	asparagine synthetase	*ASN3*	*Zm00001eb013430*	overexpression	[70]
		*ASN4*	*Zm00001eb396990*		
*SUT1*	carrier protein	*SUT1*	*Zm00001eb005460*	overexpression	[64]
		*SUT7*	*Zm00001eb402200*		
*AAP6*	carrier protein	*AAAP21*	*Zm00001eb145670*	overexpression	[65]
*NAC100*	transcription factor	*NACTF32*	*Zm00001eb080700*	knockout	[66]

**Table 4:** List of potential gene resources for enhancing vitamin contents in maize.

Genes	Protein Function	Maize Orthologs	Gene ID	Strategy	References
*DXS*	1-deoxyxylulose 5-phosphate synthase	*DXS1*	*Zm00001eb287860*	overexpression	[89]
*DXR*	1-deoxy-D-xylulose 5-phosphate reductoisomerase	*DXR1*	*Zm00001eb126690*	overexpression	[72]
		*DXR2*	*Zm00001eb334370*		
*PDS*	phytoene desaturase	*VP5*	*Zm00001eb006300*	overexpression	[90]
*OR*	coactivator		*Zm00001eb249060*	overexpression	[73,74]
*DET1*	transcription factor		*Zm00001eb317230* *Zm00001eb341540*	knockout	[75,76]
*BZR1*	transcription factor	*BES1*	*Zm00001eb325550*	overexpression	[77]
*CMB1*	transcription factor	*ZMM6*	*Zm00001eb036590*	overexpression	[78]
		*ZMM7*	*Zm00001eb317770*		
		*ZMM27*	*Zm00001eb102450*		
*SGR1*	magnesium dechelatase	*NYE1*	*Zm00001eb319560*	knockout	[79]
		*NYE2*	*Zm00001eb103480*		
*CCD4*	carotenoid cleavage dioxygenase	*NCED6*	*Zm00001eb188280*	knockout	[80,81]
		*NCED8*	*Zm00001eb251990*		
*LOX1*	lipoxygenase	*LOX4*	*Zm00001eb054050*	knockout	[82]
		*LOX5*	*Zm00001eb216870*		
*CLD1*	chlorophyll dephytylase	*UMC2173*	*Zm00001eb349130*	overexpression	[91]
*CHLG*	chlorophyll synthase	*CHLG1*	*Zm00001eb286140*	knockout	[92]
*GPPD*	ρ-hydroxyphenylpyruvate dioxygenase	*HPPD1*	*Zm00001eb232960* *Zm00001eb304950*	overexpression	[93]
*HPT*	homogentisate phytyltransferase	*HPT1*	*Zm00001eb389370*	overexpression	[94]
*HGGT*	homogentisic acid geranylgeranyl transferase	*HGGT1* *HGGT2* *HGGT3*	*Zm00001eb386720* *Zm00001eb105110* *Zm00001eb121230* *Zm00001eb382300*	overexpression	[89]
*MPBQMT*	2-methyl-6-phytylbenzoquinol methyltransferase	*APG1*	*Zm00001eb031790*	overexpression	[93]
*TC*	tocopherol cyclase	*SXD1*	*Zm00001eb237270*	overexpression	[94]
*TBP*	tocopherol-binding protein		*Zm00001eb197980* *Zm00001eb347610*	overexpression	[83]
*GULLO*	L-gulono-1,4-lactone oxidase		*Zm00001eb059530* *Zm00001eb072160* *Zm00001eb154880* *Zm00001eb236290* *Zm00001eb421440* *Zm00001eb236880*	overexpression	[95]
*GME*	GDP-mannose 3,5-epimerase	*GME1*	*Zm00001eb047980*	overexpression	[96]
		*GME2*	*Zm00001eb167750*		
*GGP*	GDP-L-galactose phosphorylase	*SI946084H12*	*Zm00001eb144410*	overexpression	[96]
*GPP*	L-galactose-1-phosphate phosphatase	*GPP1*	*Zm00001eb049310*	overexpression	[96]
*GDH*	L-galactose dehydrogenase	*GALDH1*	*Zm00001eb408730*	overexpression	[96]
*GALLDH*	L-galactono-1,4-lactone dehydrogenase	*GLDH1*	*Zm00001eb093120*	overexpression	[96]
*DHAR1*	dehydroascorbate reductase	*DHAR1*	*Zm00001eb355540*	overexpression	[85,86]
		*DHAR2*	*Zm00001eb355550*		
		*DHAR3*	*Zm00001eb266260*		
*GTPCHI*	GTP cyclohydrolase	*GCH1*	*Zm00001eb067370*	overexpression	[87]
		*GCH2*	*Zm00001eb432940*		
*ADCS*	aminodeoxychorismate synthase	*ADCS1*	*Zm00001eb272970*	overexpression	[87]
*DHFS*	dihydrofolate synthetase	*DHFS1*	*Zm00001eb410070*	overexpression	[87]
		*DHFS2*	*Zm00001eb137120*		
*FPGS*	folylpolyglutamate synthase	*FGP2* *BM4*	*Zm00001eb044170* *Zm00001eb404110* *Zm00001eb299330* *Zm00001eb421680*	overexpression	[87]
*GGH*	γ-glutamyl hydrolase		*Zm00001eb199250* *Zm00001eb353180*	overexpression	[88]

**Table 5:** List of potential gene resources for enhancing mineral contents in maize.

Genes	Protein Function	Maize Orthologs	Gene ID	Strategy	References
*NAS*	nicotianamine synthase	*NAS1*	*Zm00001eb396230*	overexpression	[98]
		*NAS2*	*Zm00001eb014700*		
		*NAS3*	*Zm00001eb052890*		
		*NAS4*	*Zm00001eb218440*		
		*NAS6*	*Zm00001eb396110*		
		*NAS8*	*Zm00001eb396250*		
		*NAS9*	*Zm00001eb014680*		
		*NAS10*	*Zm00001eb396280*		
*NAAT*	nicotianamine aminotransferase	*NAAT1*	*Zm00001eb203230*	overexpression	[99]
		*PCO115235C*	*Zm00001eb240650*		
*NRAMP1*	carrier protein	*NRAT1*	*Zm00001eb224770*	overexpression	[120]
*NRAMP5*	carrier protein	*NRAT5*	*Zm00001eb304610*	overexpression	[121]
*FRD3*	carrier protein	*MATE1*	*Zm00001eb261140* *Zm00001eb143800* *Zm00001eb424530*	overexpression	[122]
*FRDL1*	carrier protein	*MATE3*	*Zm00001eb008790*	overexpression	[123]
*VIT1*	carrier protein		*Zm00001eb424350* *Zm00001eb099160* *Zm00001eb312010*	knockout	[100]
*NRAMP3*	carrier protein	*NRAT3* *NRAT4*	*Zm00001eb400560* *Zm00001eb030050* *Zm00001eb051790*	overexpression	[101]
*FER*	storage protein	*FER1*	*Zm00001eb195010*	overexpression	[102]
		*FER2*	*Zm00001eb404870*		
*IRO2*	transcription factor	*BHLH54*	*Zm00001eb362800*	overexpression	[103]
		*BHLH126*	*Zm00001eb140680*		
*BHLH58*	transcription factor	*BHLH118*	*Zm00001eb289490*	overexpression	[104]
*BHLH59*	transcription factor	*BHLH128*	*Zm00001eb209480*	overexpression	[104]
		*BHLH129*	*Zm00001eb229950*		
*BHLH29*	transcription factor	*BHLH100*	*Zm00001eb420910*	overexpression	[105]
		*BHLH101*	*Zm00001eb085690*		
*BHLH300*	transcription factor	*BHLH54*	*Zm00001eb362800*	overexpression	[106]
*IDEF1*	transcription factor	*ABI47*	*Zm00001eb198710*	overexpression	[107]
		*ABI49*	*Zm00001eb259870*		
*CSN6*	coactivator	*SI605023C06B*	*Zm00001eb199540* *Zm00001eb034040*	knockout	[108]
*RAB6A*	small GTPase	*IDP871*	*Zm00001eb006940*	overexpression	[109]
*HRZ*	Fe-binding ubiquitin ligase	*541975*	*Zm00001eb360580* *Zm00001eb156300* *Zm00001eb294920*	knockout	[110]
*MTP1*	carrier protein	*UMC2311*	*Zm00001eb265000* *Zm00001eb385520* *Zm00001eb420140* *Zm00001eb354910*	overexpression	[111]
*ZIF1*	carrier protein	*MFSD1* *MFSD2* *IDP8516* *TOM3* *UMC1028* *IDP7324* *YS3* *IDP6979*	*Zm00001eb129050* *Zm00001eb196170* *Zm00001eb038000* *Zm00001eb093430* *Zm00001eb093440* *Zm00001eb128730* *Zm00001eb133440* *Zm00001eb163460* *Zm00001eb129340* *Zm00001eb196180* *Zm00001eb332620*	overexpression	[112]
*ZIF2*	carrier protein	*PCO099415*	*Zm00001eb017730*	overexpression	[113]
		*GPM828*	*Zm00001eb017760*		
*HMA2*	carrier protein	*HMA2*	*Zm00001eb226870*	overexpression	[114]
*HMA4*	carrier protein	*HMA3*	*Zm00001eb095020*	overexpression	[115]
*HMA7*	carrier protein	*CSU904*	*Zm00001eb327860*	overexpression	[116]
*ZIP1*	carrier protein		*Zm00001eb139810*	overexpression	[117]
*ZIP8*	carrier protein	*ZIP8*	*Zm00001eb303800*	knockout	[118]
*MIPS*	myo-inositol-1-phosphate synthase	*MIPS2*	*Zm00001eb401220* *Zm00001eb276490* *Zm00001eb283250* *Zm00001eb378070*	knockout	[124]
*PGK1*	2-phosphoglycerate kinase		*Zm00001eb191270* *Zm00001eb259060*	knockout	[125]
*ITPK2*	inositol 1,3,4-trisphosphate 5/6-kinase		*Zm00001eb399350*	knockout	[126]
*IPK1*	inositol 1,3,4,5,6-pentakisphosphate 2-kinase	*IDP8938*	*Zm00001eb067500* *Zm00001eb432760*	knockout	[127]
*HAD1*	phytase		*Zm00001eb063350* *Zm00001eb342820* *Zm00001eb399750*	overexpression	[128]
*PAPHY-A*	phytase	*PAP2*	*Zm00001eb064450*	overexpression	[129]
*PAP4*	phytase	*PAP22*	*Zm00001eb048820*	overexpression	[130]

**Table 6:** List of potential gene resources for enhancing anthocyanin content in maize.

Genes	Protein Function	Maize Orthologs	Gene ID	Strategy	References
*GLK1*	transcription factor	*G2*	*Zm00001eb118900*	overexpression	[133]
		*GLK1*	*Zm00001eb371980*		
*AN1*	transcription factor	*IN1*	*Zm00001eb303250*	overexpression	[134]
*AN3*	transcription factor	*GIF1*	*Zm00001eb056300*	overexpression	[135]
*ANT1*	transcription factor	*PL1*	*Zm00001eb278680*	overexpression	[136]
		*C1*	*Zm00001eb373660*		
*MYBR*	transcription factor	*MYB31*	*Zm00001eb103730*	knockout	[137]
		*MYB42*	*Zm00001eb202770*		
*DRB3*	double stranded RNA binding protein	*IDP7470*	*Zm00001eb102530*	knockout	[138]

## Data Availability

The data presented in this study are available in Table S1, Figures S1 and S2.

## Supplementary Materials

The following supporting information can be downloaded at: https://www.mdpi.com/article/10.3390/plants11050627/s1, Figure S1: Chromosomal locations of potential gene resources; Figure S2: Expression profiles of potential gene resources for maize; Table S1: Expression levels of 246 maize potential gene resources for nutrient improvement among the different tissues.
