# Supplementary material for: Mining of Potential Gene Resources for Breeding Nutritionally Improved Maize
Source: Plants (Basel). 2022 Feb 25;11(5):627. doi: 10.3390/plants11050627 (PMC8912576; doi:10.3390/plants11050627)
Supplement: Supplementary file 1 [file plants-11-00627-s001.zip › plants-1577955-supplementary/Figure S1.pdf]

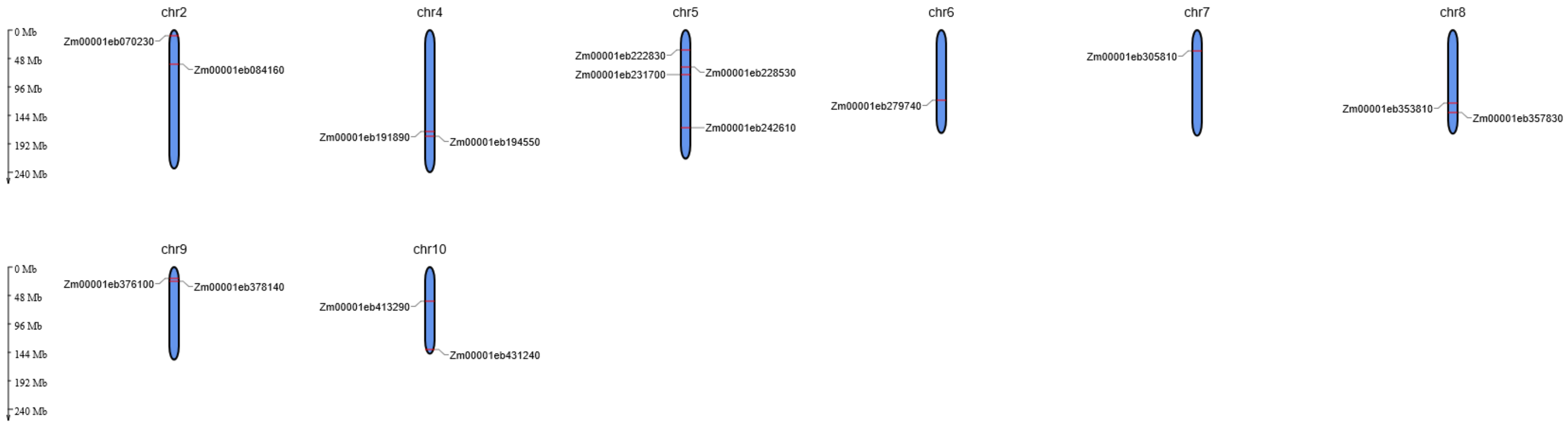

Figure S1-1. Chromosomal locations of potential gene resources for improving resistant starch content in maize. The scale is in megabases (Mb).

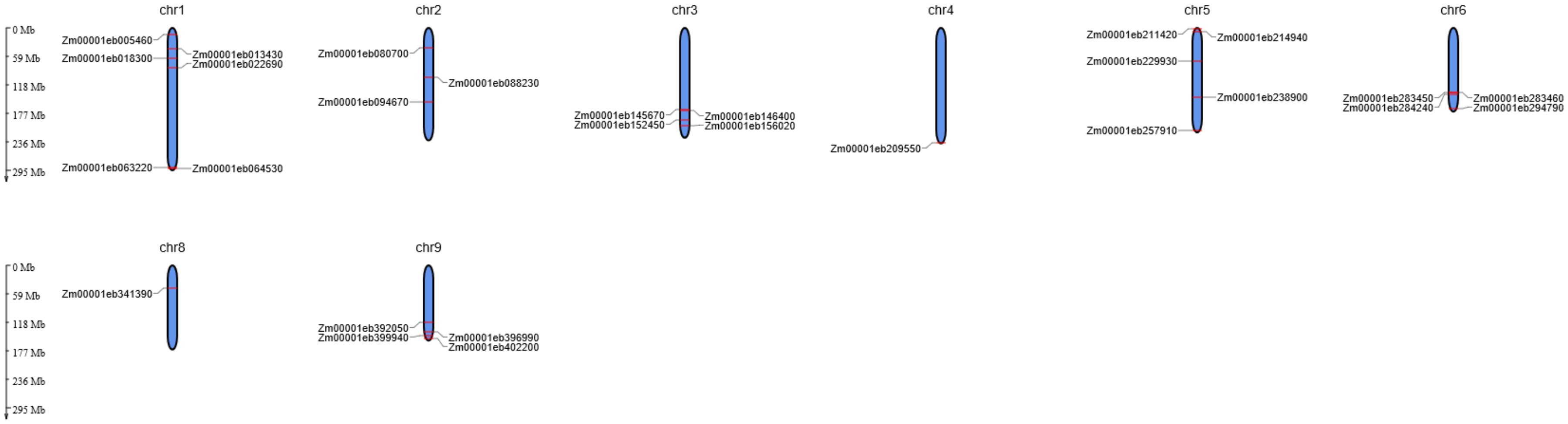

Figure S1-2. Chromosomal locations of potential gene resources for elevating essential amino acid content in maize. The scale is in megabases (Mb).

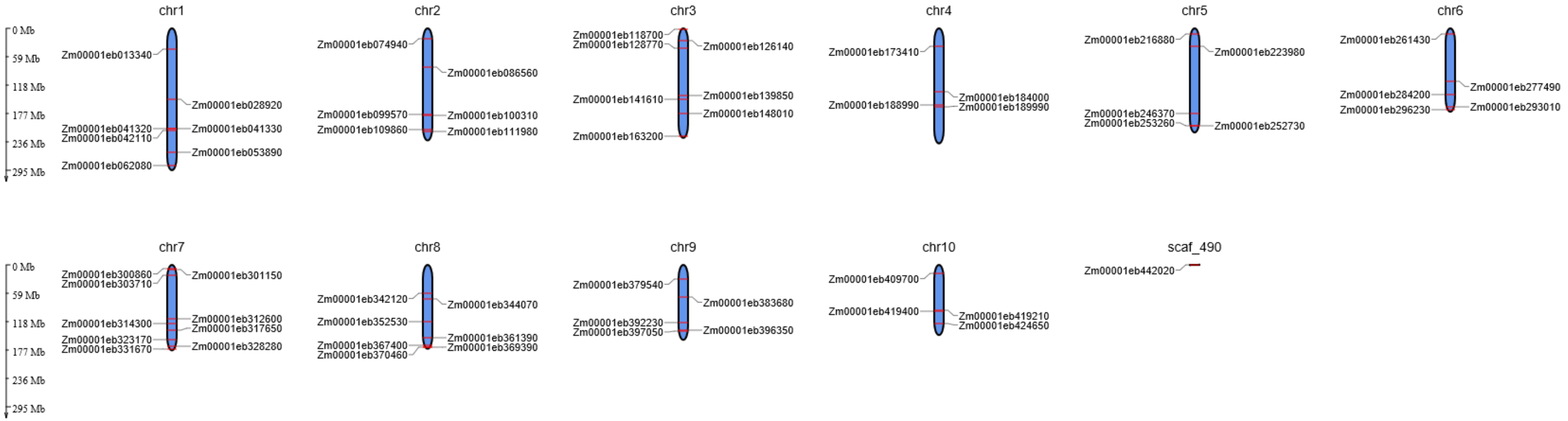

Figure S1-3. Chromosomal locations of potential gene resources for enhancing lipid yield in maize. The scale is in megabases (Mb).

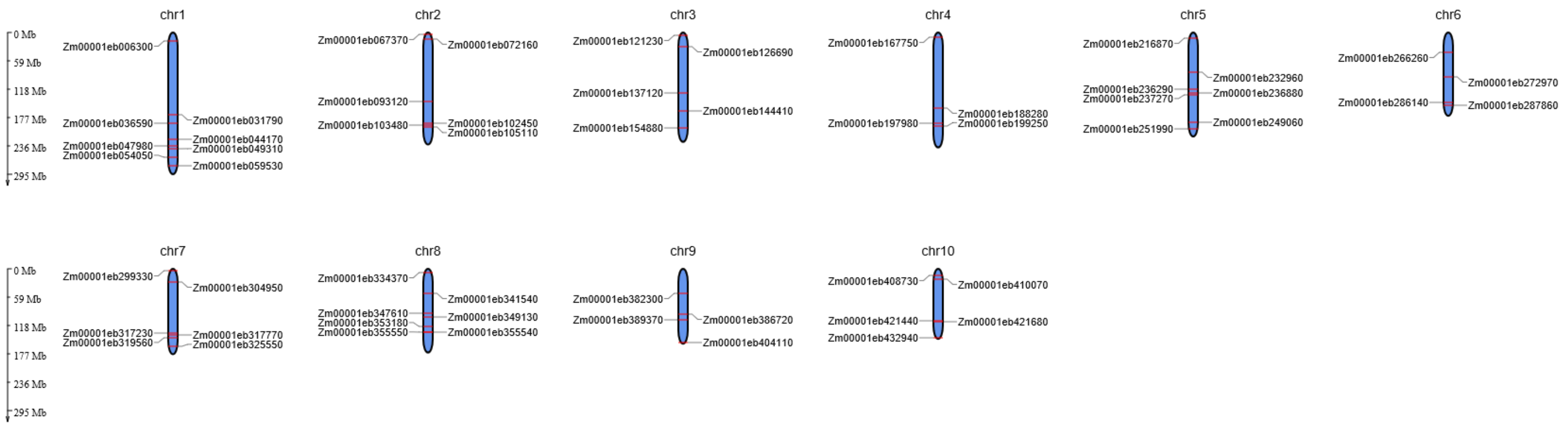

Figure S1-4. Chromosomal locations of potential gene resources for enhancing vitamin contents in maize. The scale is in megabases (Mb).

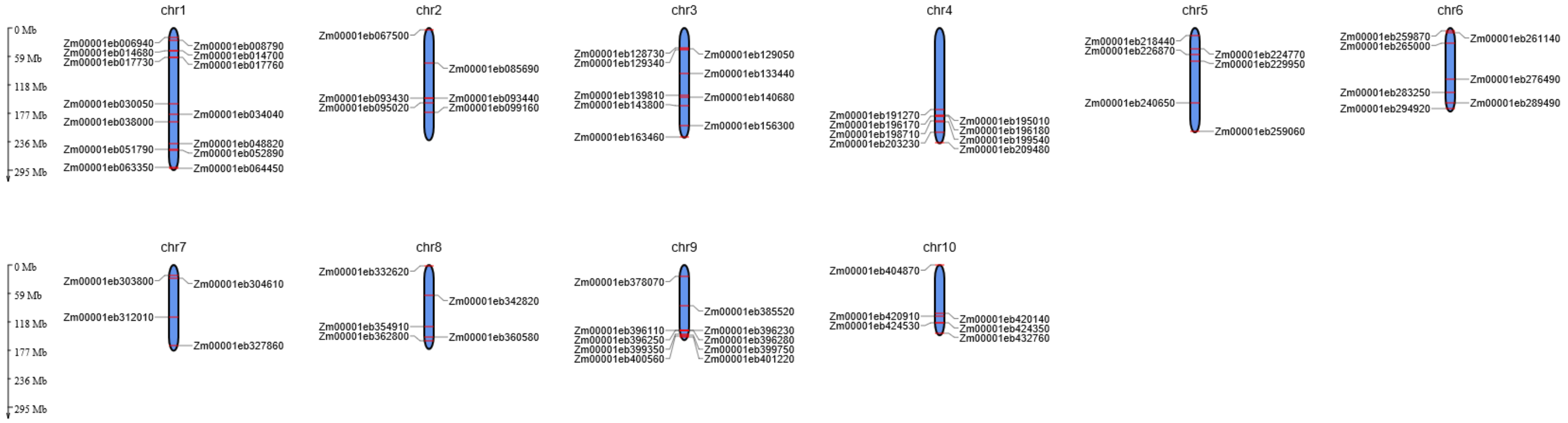

Figure S1-5. Chromosomal locations of potential gene resources for enhancing mineral contents in maize. The scale is in megabases (Mb).

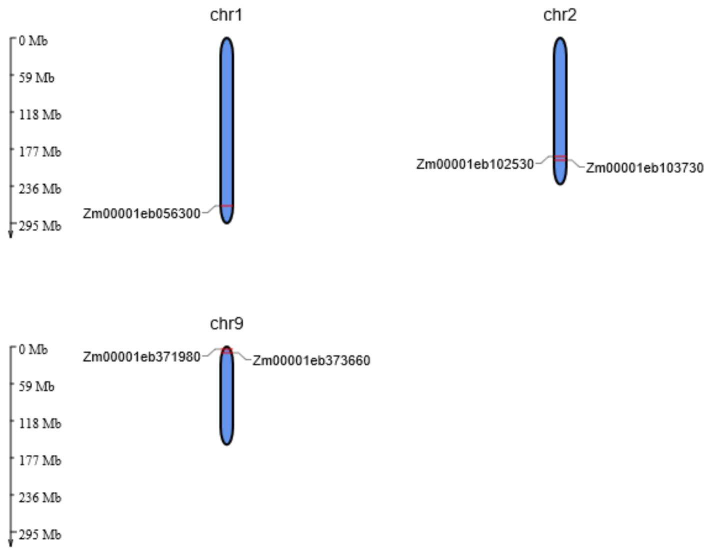

Figure S1-6. Chromosomal locations of potential gene resources for enhancing anthocyanin content in maize. The scale is in megabases (Mb).
