# Supplementary material for: Mining of Potential Gene Resources for Breeding Nutritionally Improved Maize
Source: Plants (Basel). 2022 Feb 25;11(5):627. doi: 10.3390/plants11050627 (PMC8912576; doi:10.3390/plants11050627)
Supplement: Supplementary file 1 [file plants-11-00627-s001.zip › plants-1577955-supplementary/Figure S2.Expression profiles of potential gene resources for maize.pdf]

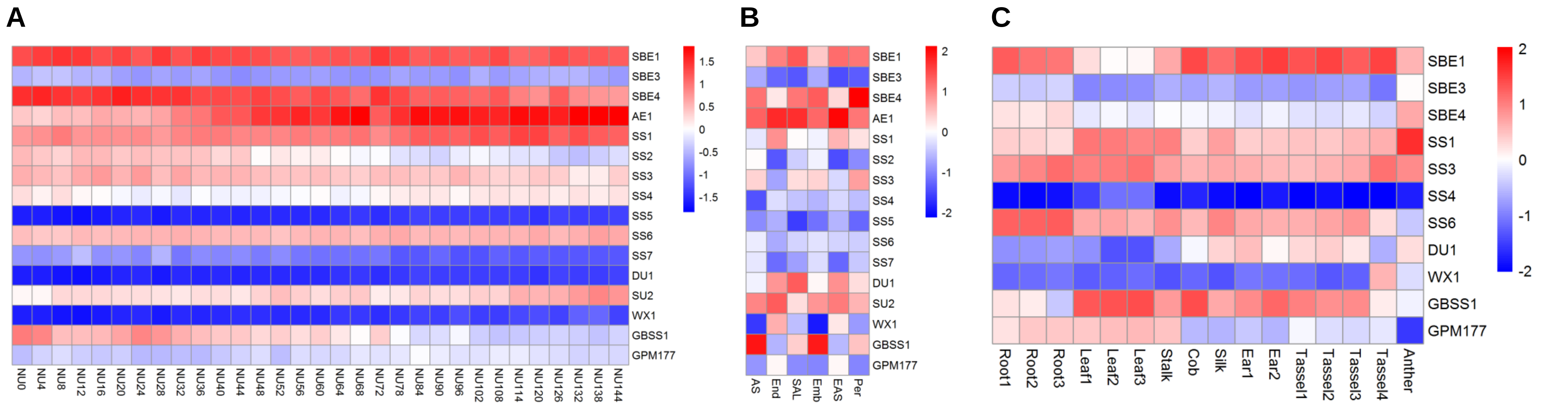

Figure S2-1. Expression profiles of potential gene resources for maize starch content improvement. (A) Expression heatmap of potential gene resources for maize starch content improvement from maize nucellus at different time points after pollination. NU0-144 represents the nucellus (embryo sac included) after 0-144 hours of self-pollination. (B) Expression heatmap of potential gene resources for maize starch content improvement from maize kernels. AS, Apical scutellum; End, Endosperm; SAL, Scutellar Alleurone Layer; Emb, Embryo; EAS, Endosperm Adjacent to Scutellum; Per, Pericarp. (C) Expression heatmap of potential gene resources for maize starch content improvement from 16 maize tissues. Root1,2,3 represent V2, V5 seminal root and adult nodal root, respectively; Leaf1,2,3 represent the 2nd, 4th and 8th leaf, respectively; Ear1,2 represent V8 and V15 ear, respectively; Tassel 1,2,3,4 represent 1mm, 2cm, 12cm and 22cm tassel, respectively. The color scale bars represent the relative expression level.

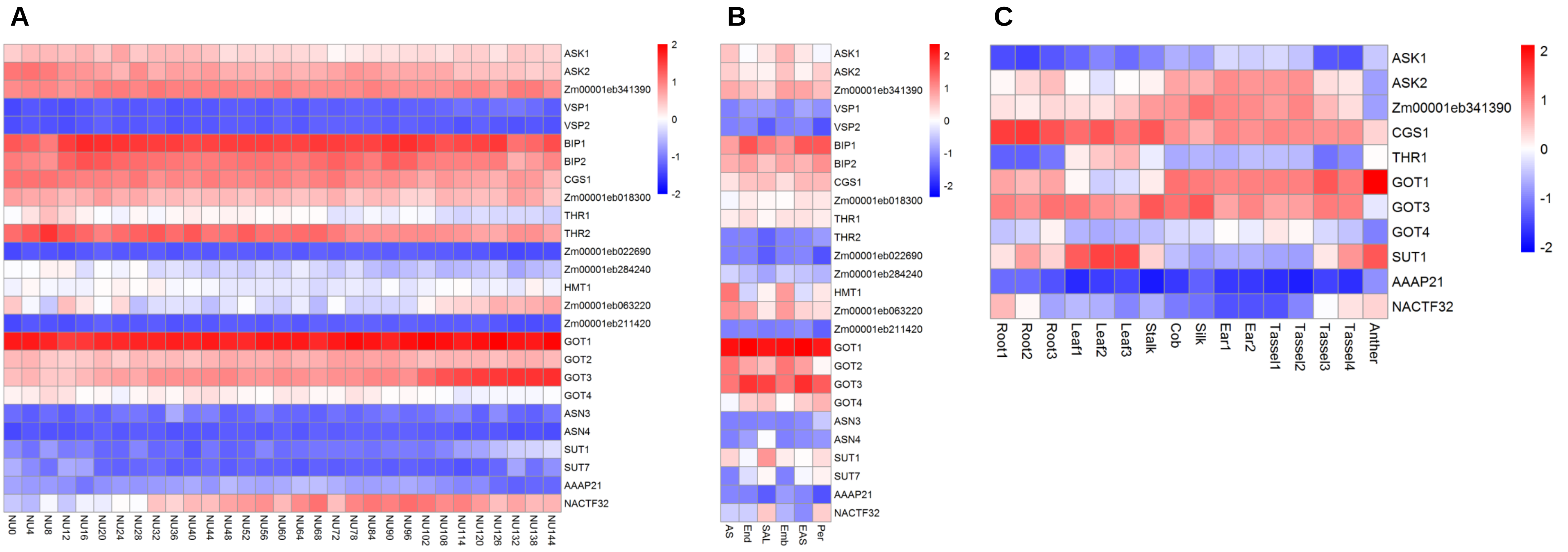

Figure S2-2. Expression profiles of potential gene resources for maize essential amino acid content improvement. (A) Expression heatmap of potential gene resources for maize essential amino acid content improvement from maize nucellus at different time points after pollination. (B) Expression heatmap of potential gene resources for maize essential amino acid content improvement from maize kernels (C) Expression heatmap of potential gene resources for maize essential amino acid content improvement from 16 maize tissues. Annotations for tissue abbreviations are the same as in Figure S2-1. The color scale bars represent the relative expression level.

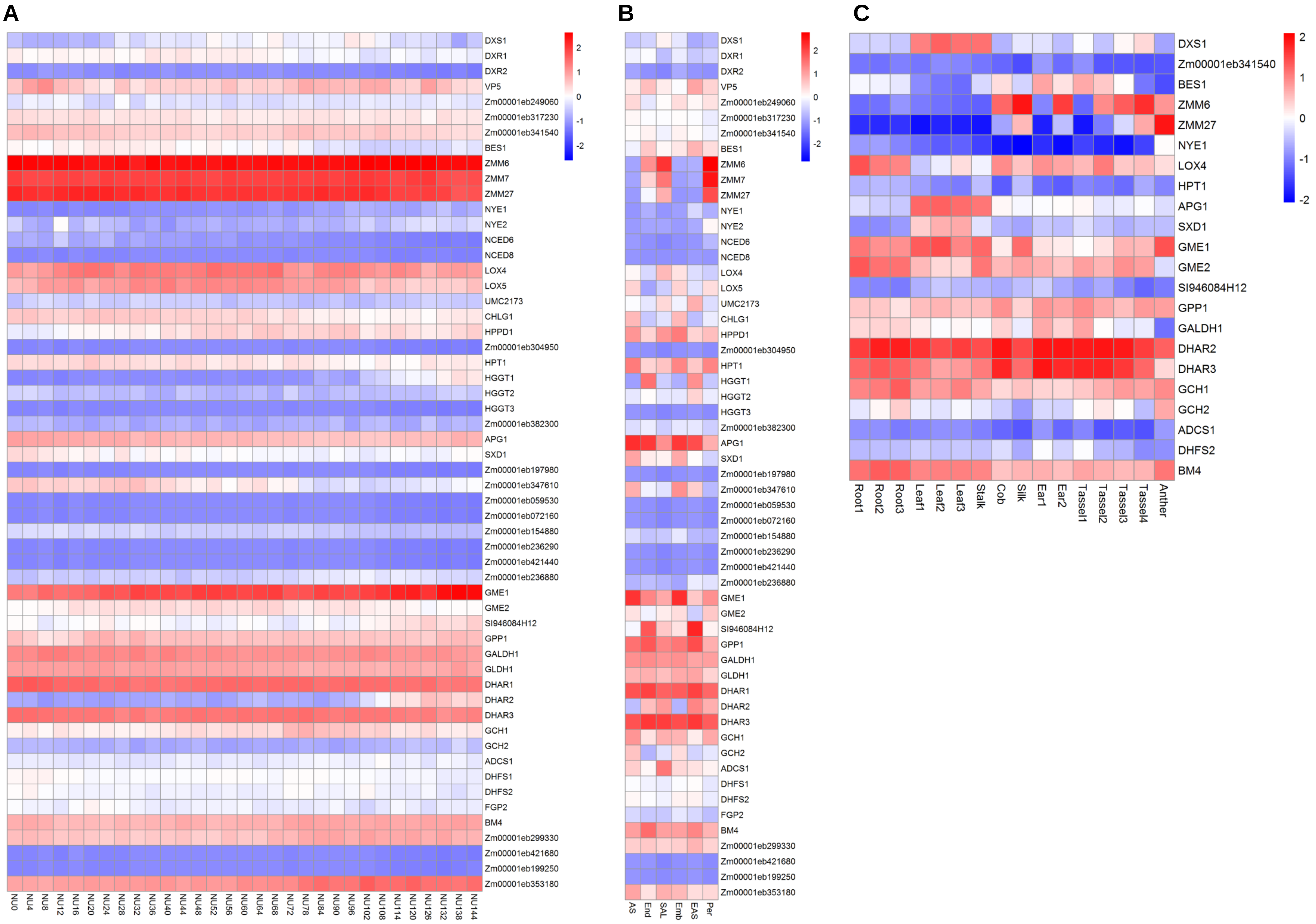

Figure S2-3. Expression profiles of potential gene resources for maize vitamin content improvement. (A) Expression heatmap of potential gene resources for maize vitamin content improvement from maize nucellus at different time points after pollination. (B) Expression heatmap of potential gene resources for maize vitamin content improvement from maize kernels. (C) Expression heatmap of potential gene resources for maize vitamin content improvement from 16 maize tissues. Annotations for tissue abbreviations are the same as in Figure S2-1. The color scale bars represent the relative expression level.

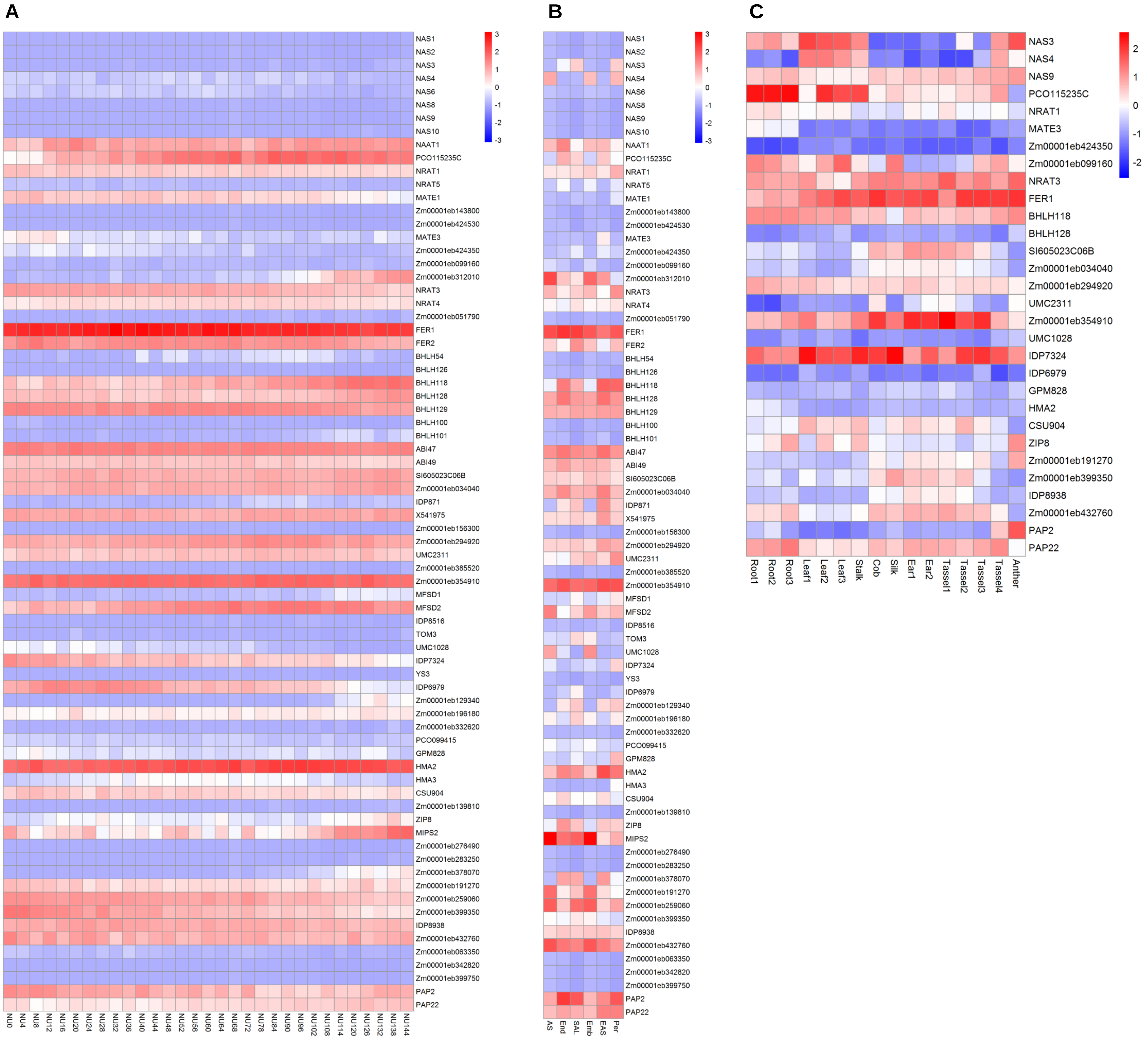

Figure S2-4. Expression profiles of potential gene resources for maize mineral content improvement. (A) Expression heatmap of potential gene resources for maize mineral content improvement from maize nucellus at different time points after pollination. (B) Expression level heatmap of potential gene resources for maize mineral content improvement from maize kernels. (C) Expression heatmap of potential gene resources for maize mineral content improvement from 16 maize tissues. Annotations for tissue abbreviations are the same as in Figure S2-1. The color scale bars represent the relative expression level.

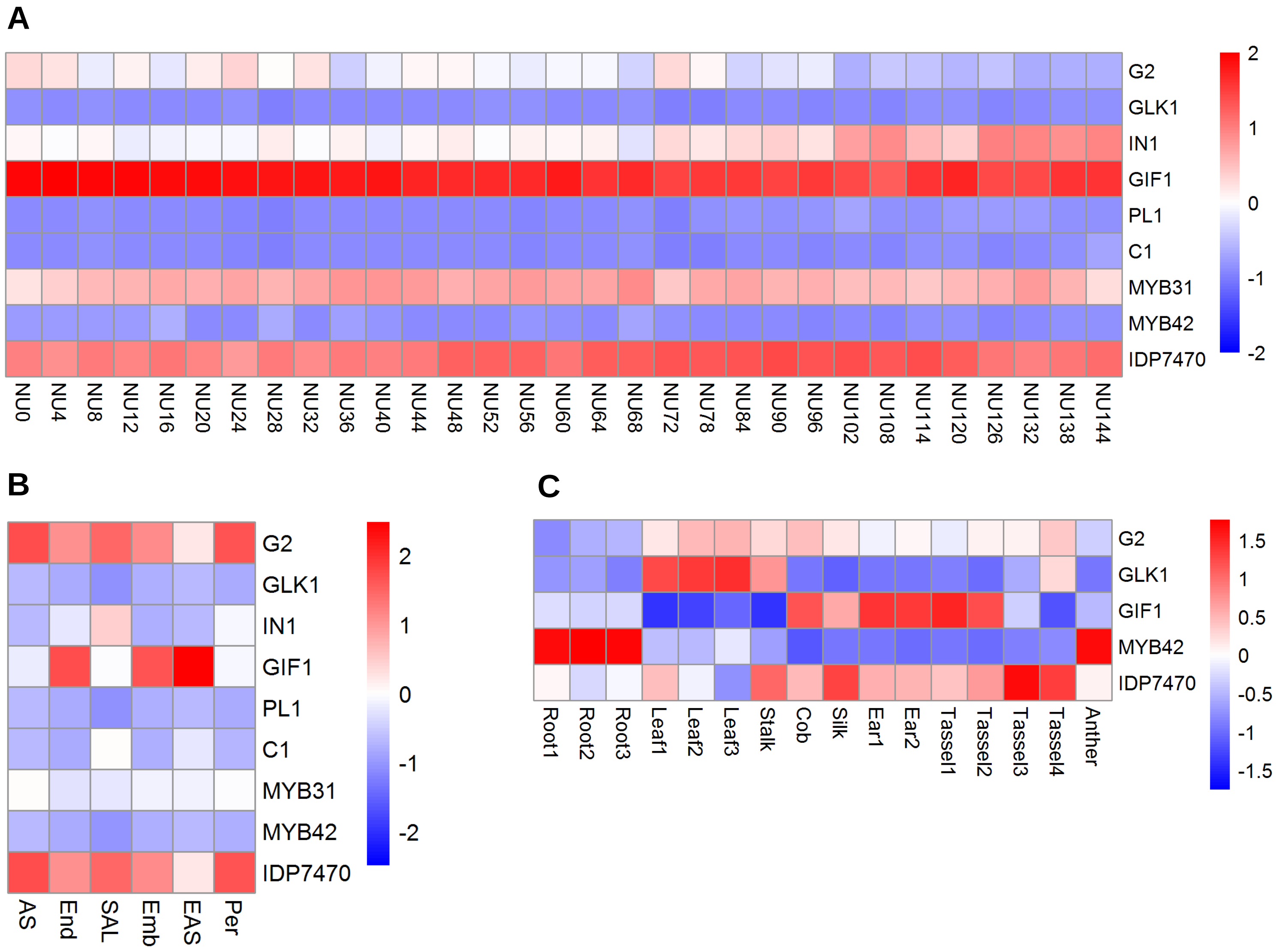

Figure S2-5. Expression profiles of potential gene resources for maize anthocyanin content improvement. (A) Expression heatmap of potential gene resources for maize anthocyanin content improvement from maize nucellus at different time points after pollination. (B) Expression heatmap of potential gene resources for maize anthocyanin content improvement from maize kernels. (C) Expression heatmap of potential gene resources for maize anthocyanin content improvement from 16 maize tissues. Annotations for tissue abbreviations are the same as in Figure S2-1. The color scale bars represent the relative expression level.
